# Supplementary material for: Indigenous Ancestry and Admixture in the Uruguayan Population
Source: Front Genet. 2021 Sep 23;12:733195. doi: 10.3389/fgene.2021.733195 (PMC8495321; doi:10.3389/fgene.2021.733195)
Supplement: Supplementary file 3 [file DataSheet1.pdf]

Contents

Section 1: Supplementary Figures
Section 2: Supplementary Methods

Section 1: Supplementary Figures

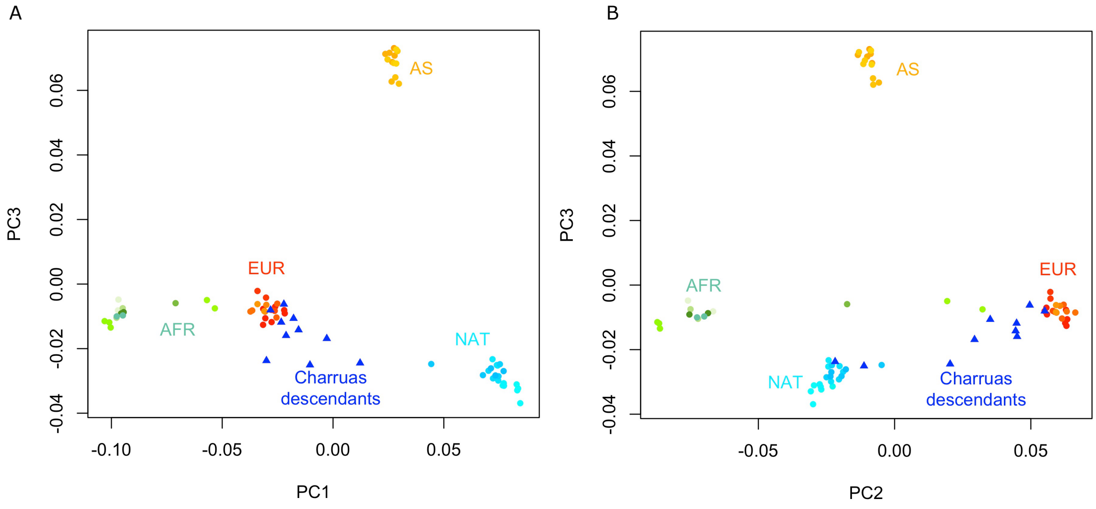

Figure S1: Higher PCs. **A.** PC1 vs PC3 **B.** PC2 vs PC3

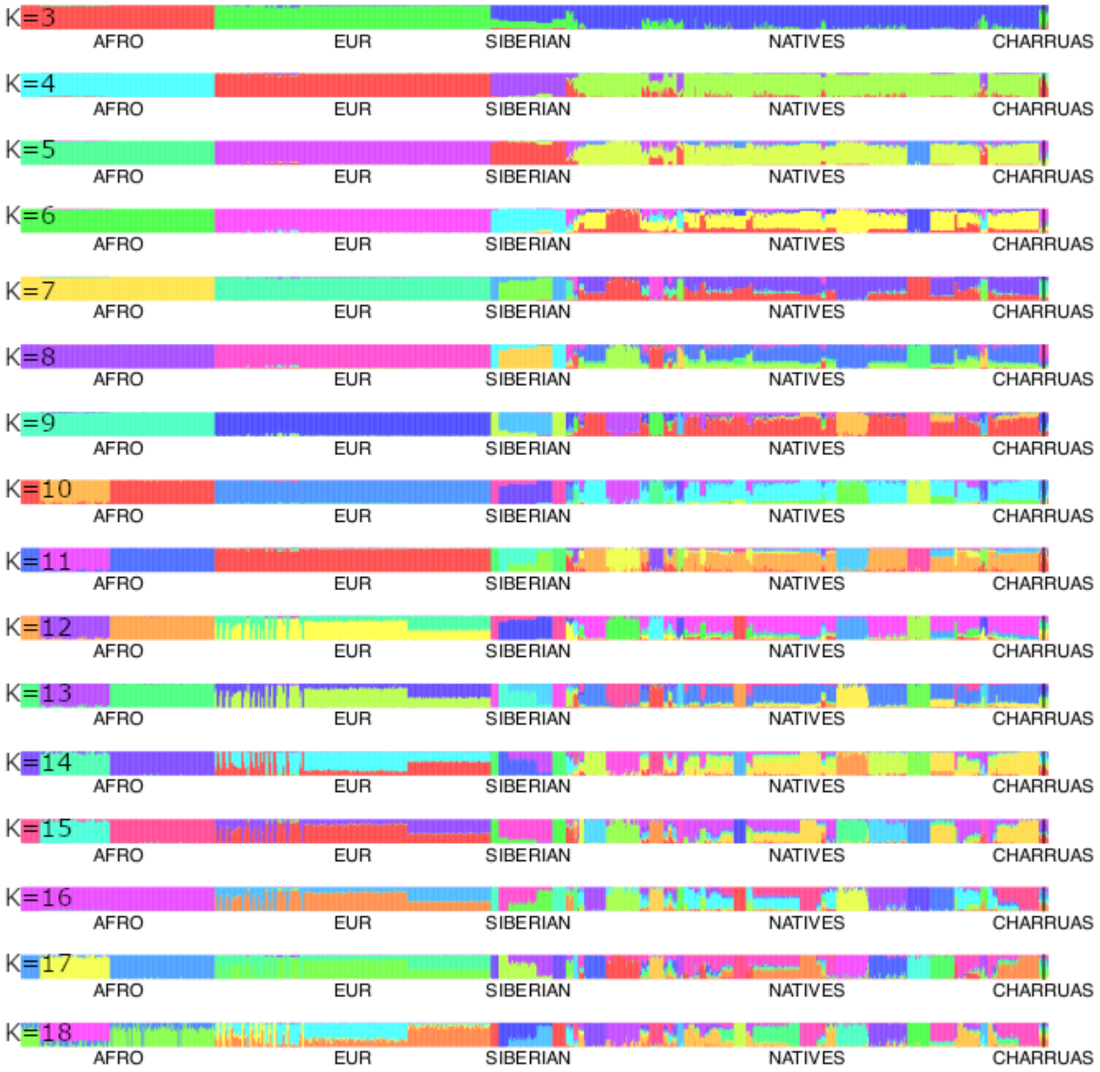

Figure S2: Admixture with higher K. K ranges from K=3 to K=18. To the left all individuals from the reference panel. To the right only the Charrúa descendants.

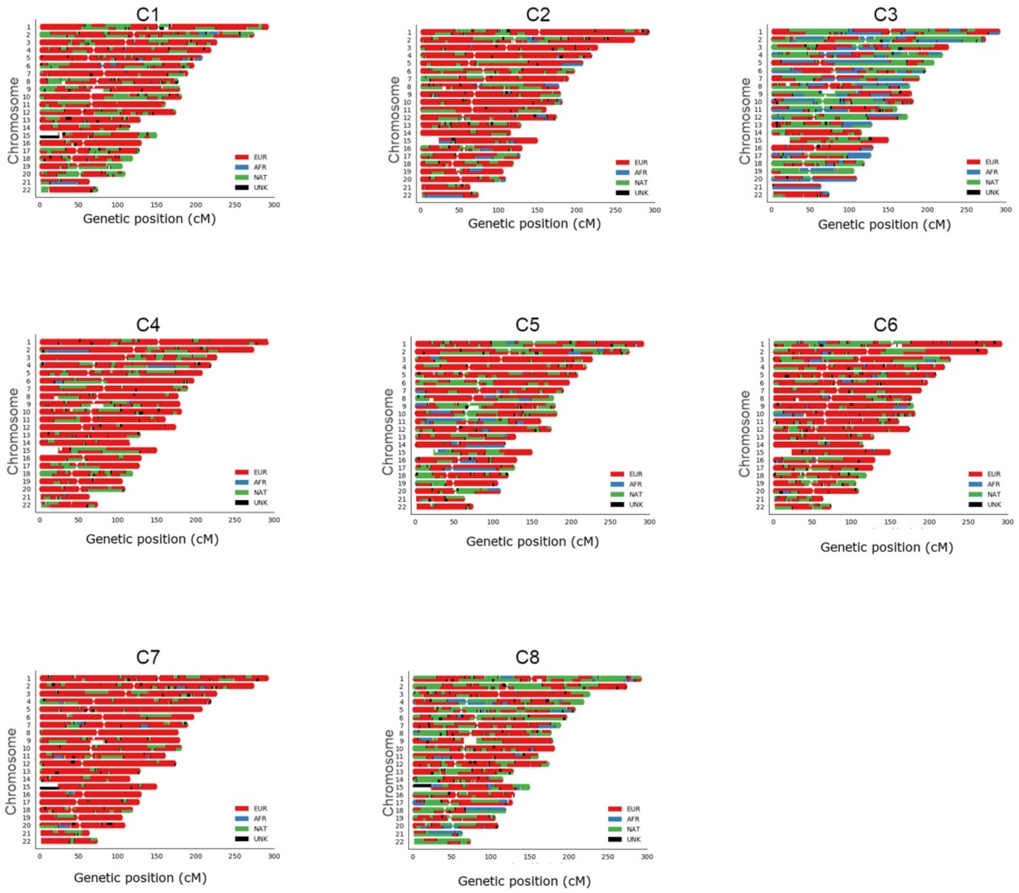

Figure S3: Karyograms resulting from RFMix. Karyogram for all individuals as resulted from the RFMix run.

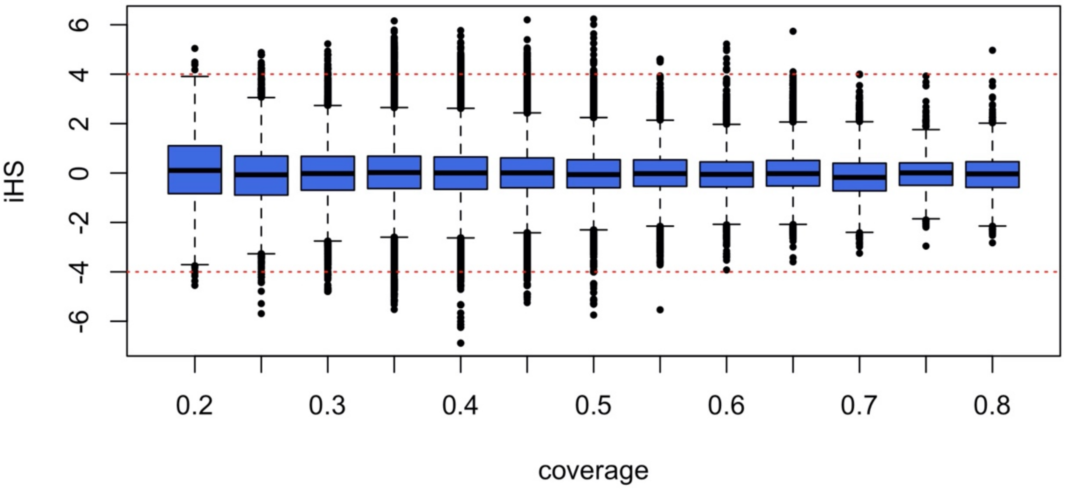

Figure S4: Indigenous haplotypes compared to iHS values. For each proportion of indigenous ancestry covering each position in the genome we determined the distribution of his values.

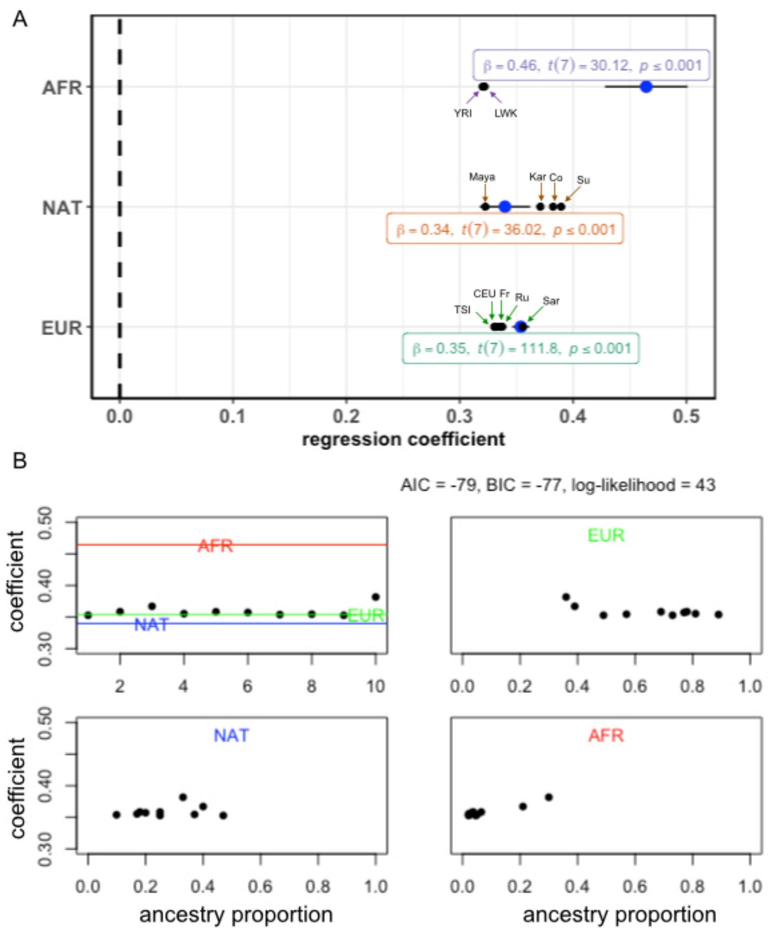

Figure S5: Heterozygosity. A. Regression coefficient for three ancestries: AFR, EUR and NAT. B. First panel, regression coefficient for each individual. The other panels show the ancestry proportions against the regression coefficient for each ancestry separately. YRI: Yoruba, LWK: Luhya in Webuye, Kenya, Co: Colombia, Kar: Karitiana, Su: Surui, CEU: Northern/Central European, Fr: France, Sar: Sardinian, TSI: Toscana, Italy, Ru: Russia

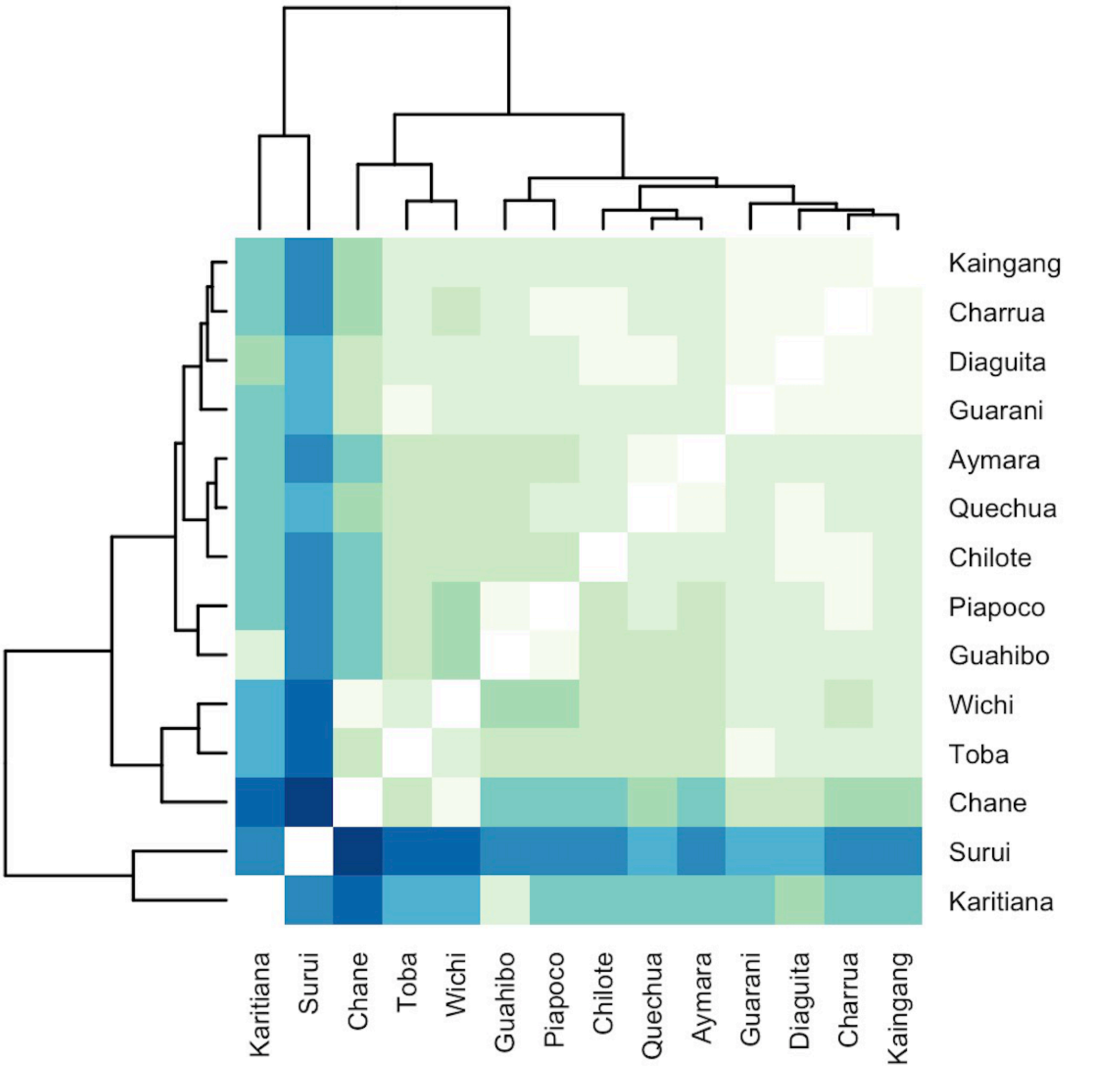

Figure S6: Fst heatmap. Heatmap representing the pairwise F_ST values for Amerindian populations.

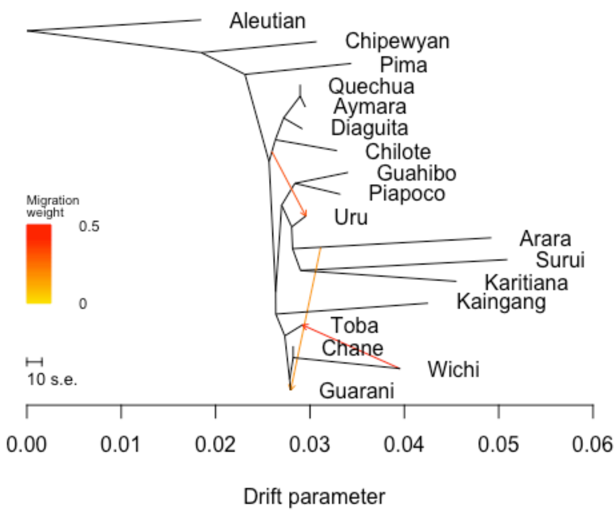

Figure S7. Treemix. Results of the Treemix algorithm applied to the Uruguayan descendants and the set of available indigenous groups. Here, we used 18 population groups: Northern populations Aleutian, Chipewyan and Pima were used to properly root the tree, the rest are South-American tribes. Mixe, Mixtec, Cabecar and Tepehuano were excluded from the analysis in order not to generate long branches that would difficult the interpretation of the South American group.

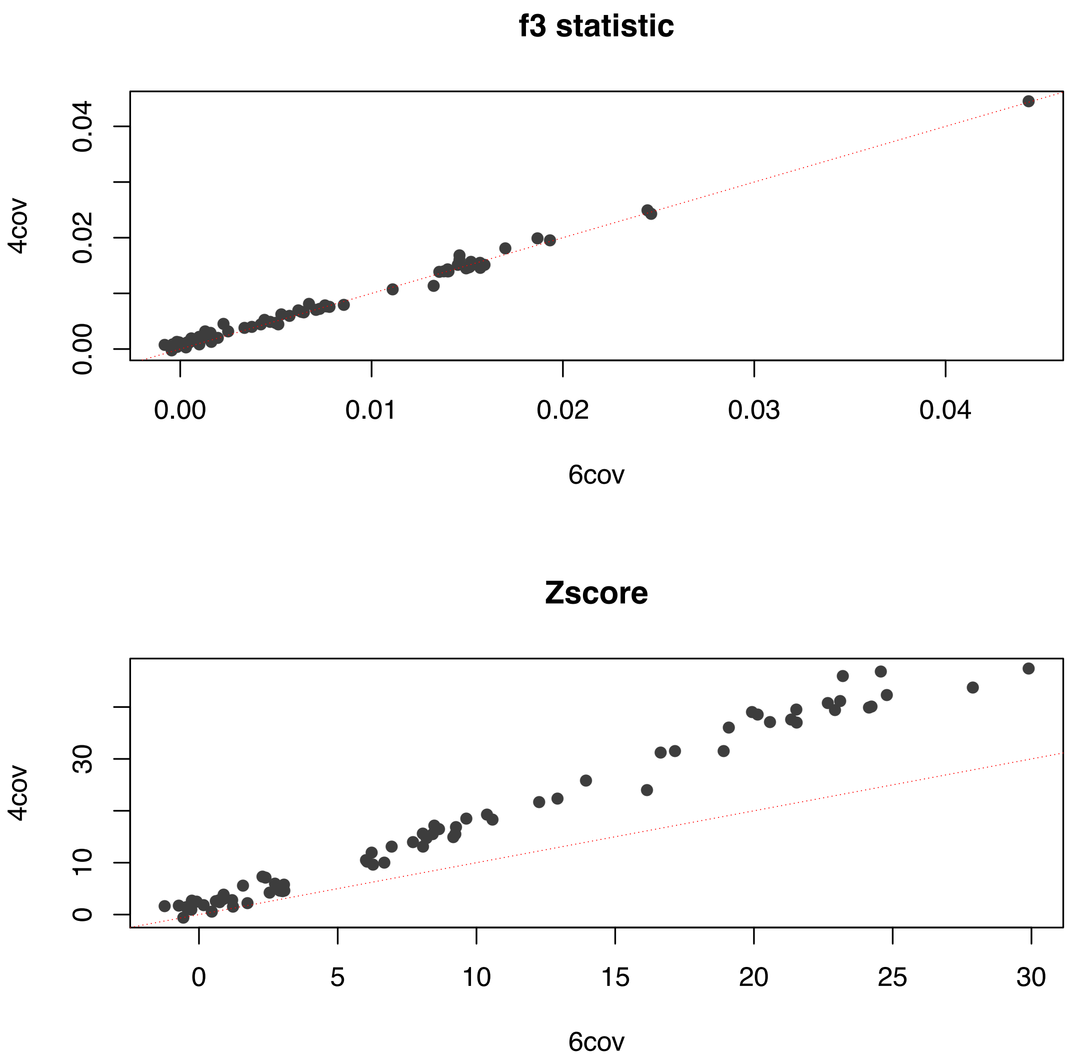

Figure S8. F3 statistics compared to indigenous haplotype coverage. In the top panel F3 statistics values as calculated in positions with 6 and 4 indigenous haplotypes. IN the bottom panel the Z-score of such calculations.

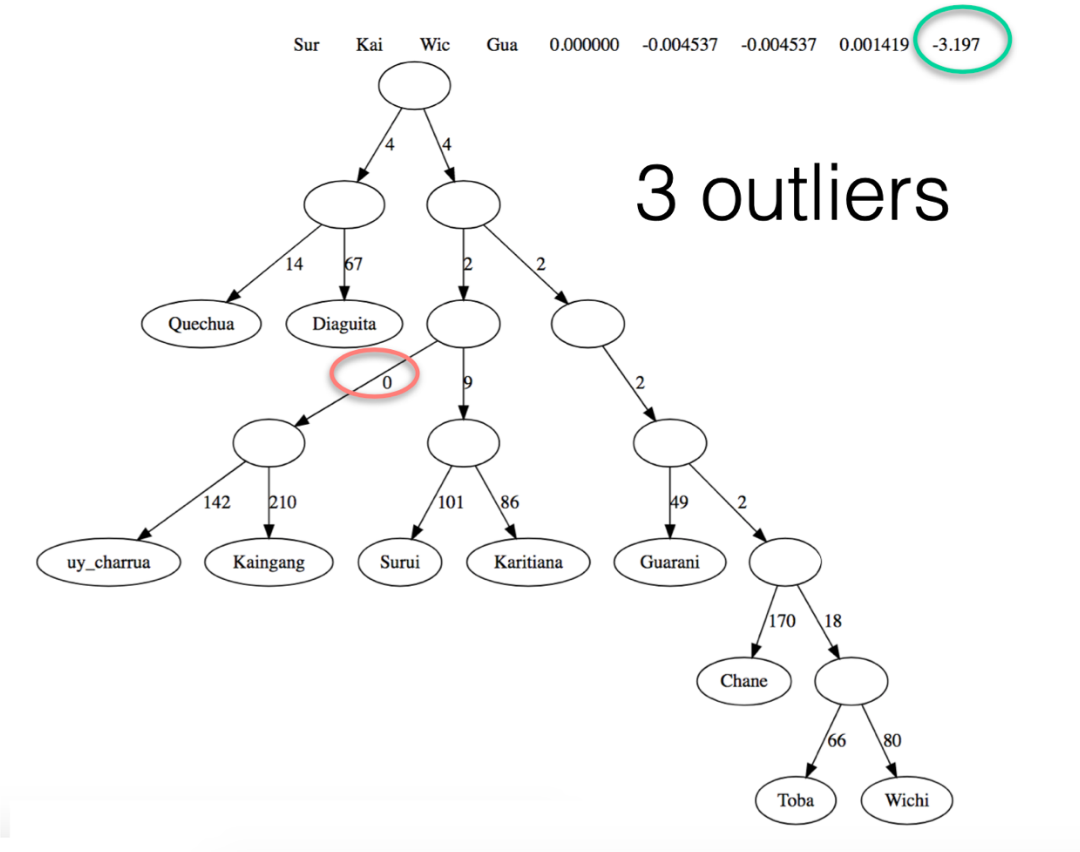

Figure S9. Alternative topology of qpGraph.

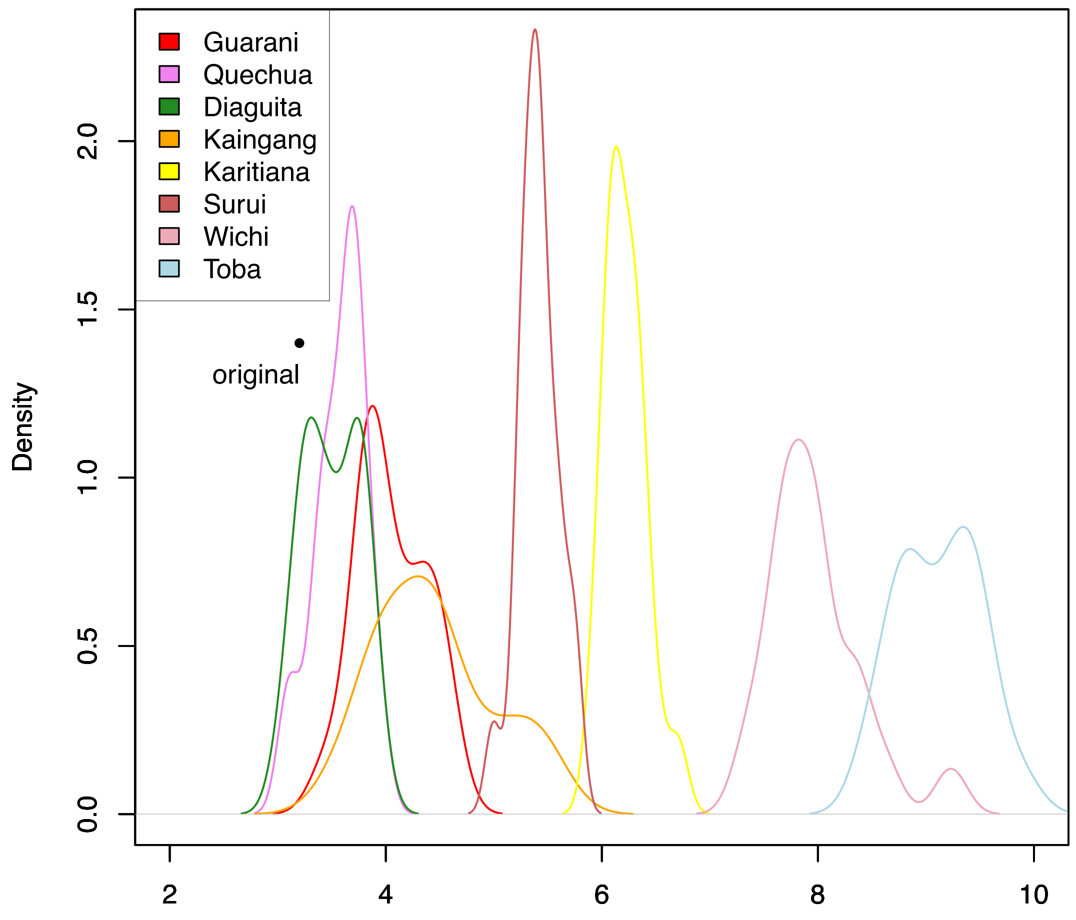

Figure S10. Z-score of simulated data. In turn, each population were masked out (50%) and pseudohaploids were constructed. Then, qpgraph was run on that data set. Distribution of Z-scores are shown in different colors corresponding to the masked population. Black point correspond to the original value with the complete ("full") data set.

<u>Section 2: Supplementary Methods</u>

<u>Supplementary Material S1: Native tracts determination with different reference panels</u>

RFMix was used for native tracts determination [1]. Different sets of parameters were used in order to compare the incidence of different reference panels and address robustness of our estimations. Four different runs of RFMix were made and two of ADMIXTURE.

First, estimations using the SNP microarray data of [2] were done. In this chip 169 Native Americans were genotyped at 364,470 single nucleotide polymorphisms, together with European and Africans, among others. This set of Europeans, Africans and Natives was taken as reference panel for native tracts determination of the Uruguayan genomes, which were subset at those chips positions. Proportions of each ancestry estimation is given in Supplementary Table S3 (tab RFMix_chip).

In order to have a finer estimation of the tracts genome-wide other reference panels were used. This second approach uses 21 Native American whole genomes as kindly obtained from the Simons Foundation Project [3] together with 20 Africans and 21 Europeans of the 1000Genomes project (tab RFMix_WGS_1).

Third, we changed the reference panel to a subset of individuals of the 1000Genomes, using PEL (Peruvians of Lima) as the native population (tab RFMix_WGS_2). These individuals are the ones having the largest native contribution of that project.

Fourthly, we used one IBS and one YRI individuals from the 1000G and one Anzick genome (tab RFMix_WGS_3).

Lastly, ADMIXTURE proportions (K=3) using the chip data set as reference panel (tab ADMIXTURE_chip) and whole genomes of Simon (tab_ADMIXTURE).

Proportions are relatively stable among the different approaches. Regarding the three RFMix runs with Simons data, PEL or Anzick, the later was largely the most different, probably underestimating Native fractions and overestimating European. The median difference between Simons and Anzick in Native fraction was ~10% and similar for the European fraction. This might be expected since Anzick-1 is a Paleo-Indian male infant found in Montana dated to 12,707–12,556 years BP, very ancient and northern in comparison to the probable more modern and southern sample that we are analyzing. Additionally, only one genome was available from each reference population in this analysis, so that intra population variability was not addressed properly. Between PEL and Simons WGS RFMix analysis, the differences were not as large. The median difference between the native contribution was less than 3%, the median difference for the European part was little above 3% and the difference for the African ancestry was minimal. Differences are shown in S10, tab DIFFs. With PEL reference panel Native contribution could be underestimated since Peruvians are not Natives but admixed individuals from Lima, population that has only around 84% of Amerindian genes [4]. And even those native genes might have its own characteristics not shared with this population under study.

We were more confident with Simons results, since having admixed individuals in the reference panel could have some influence in the correct assignment of the tracts.

<u>Reference:</u>

[1] Maples, Brian and Gravel, Simon and Kenny, Eimear E. and Bustamante, Carlos D. (2013) RFMix: A Discriminative Modeling Approach for Rapid and Robust Local-Ancestry Inference. AJHG, 93:278–288.

[2] Reich, David and Patterson, Nick and Campbell, Desmond and Tandon, Arti and Mazieres, Stephan and et al. (2012) Reconstructing Native American Population History. Nature, 488: 370-374.
[3] Mallick, Swapan and Li, Heng and Lipson, Mark and Mathieson, et al. (2016) The Simons Genome Diversity Project: 300 genomes from 142 diverse populations. Nature, 538.
[4] Sandoval, JR and Salazar-Granara, A and Acosta, O and Castillo-Herrera, et al. (2013) Tracing the genomic ancestry of Peruvians reveals a major legacy of preColumbian ancestors. J Hu Genet, 58: 627-634.
